# EBV-associated gastric carcinoma occurring one year after metabolic bariatric surgery: case report and literature review

**DOI:** 10.3389/fonc.2026.1873529

**Published:** 2026-07-01

**Authors:** YingXin Wu, Xinxi Yang, Yuanyuan Chen, Bing Wang, Yanjun Liu, Tianqi Lu

**Affiliations:** 1 Section for Gastrointestinal Surgery, Department of General Surgery, Affiliated Hospital of Southwest Jiaotong University, The Third People’s Hospital of Chengdu, Chengdu, Sichuan, China; 2 Obesity and Metabolism Medicine-Engineering Integration Laboratory, Department of General Surgery, Affiliated Hospital of Southwest Jiaotong University, The Third People’s Hospital of Chengdu, Chengdu, Sichuan, China; 3 Department of Pathology, Affiliated Hospital of Southwest Jiaotong University, The Third People’s Hospital of Chengdu, Chengdu, Sichuan, China; 4 Center of Gastrointestinal and Minimally Invasive Surgery, Department of General Surgery, Affiliated Hospital of Southwest Jiaotong University, The Third People’s Hospital of Chengdu, Chengdu, Sichuan, China

**Keywords:** case report, Epstein–Barr virus, gastric cancer, metabolic bariatric surgery, obesity

## Abstract

Metabolic bariatric surgery (MBS) does not appear to increase the overall risk of gastric cancer; therefore, there are few documented cases of Epstein–Barr virus–associated gastric carcinoma (EBVaGC) arising early after single anastomosis sleeve ileal bypass (SASI). Here, we report a 62-year-old man who underwent SASI in July 2024 and remained asymptomatic on follow-up. Surveillance endoscopy at 12 months disclosed a 1.2 × 1.0 cm deeply excavated ulcer in the posterior gastric body; biopsy revealed adenocarcinoma with Helicobacter pylori negativity and imaging showed focal wall thickening. Indocyanine green–guided laparoscopic radical gastrectomy demonstrated moderately to poorly differentiated EBVaGC with diffuse EBER *in situ* hybridization positivity, preserved mismatch repair protein expression, wild-type p53, and a high proliferative index (Ki-67 ~90%). Targeted sequencing identified an ARID1A truncating mutation (p.Y215*, VAF 4.56%) and CCND1 amplification (~6 copies). Plasma EBV and postoperative circulating tumor DNA minimal residual disease testing were negative (single ATR VUS at 0.10%). The patient recovered uneventfully and remained disease-free at 8 months. This case highlights the biologic plausibility of tissue EBV positivity despite negative plasma EBV in EBVaGC and supports a post-MBS evaluation strategy that incorporates EBER-ISH and molecular stratification for suspicious gastric lesions, with a lower threshold for diagnostic endoscopy in patients with persistent upper gastrointestinal symptoms or unexplained nutritional abnormalities; routine gastroscopic assessment at approximately 1 year after MBS may also be considered to evaluate remnant gastric mucosal health.

## Introduction

Epstein-Barr virus (EBV) is a common and persistently infectious virus, with approximately 95% of the global population having been infected, typically resulting in asymptomatic lifelong infection (1). EBV-associated gastric carcinoma (EBVaGC) represents a distinct molecular subtype of gastric cancer. EBVaGC accounts for approximately 7.5%-10% of gastric cancer (GC) cases, with this proportion potentially reaching up to 26% in gastric stump carcinoma (2, 3). This highlights the clinical value of tissue-based EBV testing, particularly Epstein–Barr virus-encoded RNA (EBER) *in situ* hybridization, when evaluating postoperative gastric lesions with negative circulating EBV results. Current evidence suggests that metabolic bariatric surgery (MBS), as a treatment for obesity, does not increase the overall occurrence rate of gastric cancer and may even reduce the risk (4, 5). Therefore, high-quality studies on the occurrence of gastric cancer cases in the early postoperative period following MBS surgery remain scarce, especially those related to specific subtypes such as EBVaGC. The anatomical remodeling, immunometabolic reprogramming, reflux exposure, alterations in microbial communities, and nutritional fluctuations following MBS may collectively foster a pro-inflammatory and pro-methylation environment, thereby promoting the expansion and malignant transformation of EBV-latently infected epithelial clones (6–8). To date, no histopathologically confirmed cases or case series of EBVaGC following MBS have been reported. Here, we report a histopathologically confirmed case of EBVaGC occurring one year after single anastomosis sleeve ileal bypass (SASI).

## Case presentation

A 62-year-old male with a BMI of 40.19 kg/m^2^ and a 10-year history of smoking cessation presented with a history of type 2 diabetes mellitus. His blood glucose was managed through diet, exercise, and oral anti-hyperglycemic medications, yet he experienced persistent weight gain. Based on the patient’s clinical presentation and personal preference, he successfully underwent SASI in July 2024. Postoperative nutrient supplementation (multivitamins, calcium, and protein) was administered. Follow-up assessments at 1 month and 3 months postoperatively showed favorable outcomes, with all relevant metabolic parameters within normal ranges (**Table 1**). At the 6-month postoperative mark, the patient did not attend the scheduled follow-up visit as he reported feeling well and was asymptomatic.

**Table 1:** Results of monitoring patient-related metabolic parameters.

Laboratory test	Time			Reference range
	July 4, 2024^a^	October 10, 2024^b^	July 3, 2025^c^	
FPG	5.91	4.87	5.16	3.90-6.10 mmol/L
Fasting insulin	31.30↑	11.9	8.0	2.6-24.9 μU/ml
HbA1c	6.4↑	5.1	5.0	4.0-6.0%
Total cholestero	6.05↑	4.36	3.62	2.80-5.72 mmol/L
Serum ferritin	50.60	302.0	264.0	30.00-400.00 ng/ml
Serum folate	11.90	25.4	8.91	4.5-32.2 ng/ml
Vitamin B12	418	590	480	197–771 pg/ml
BMI	40.19	32.72	29.4	kg/m2

1.) “a, prior to bariatric-metabolic surgery; b, three months after bariatric-metabolic surgery; c, prior to gastric cancer surgery.” 2.) “FPG, Fasting plasma glucose; HbA1c, Glycated hemoglobin A1c; BMI, Body Mass Index.”.

**Figure 1:**
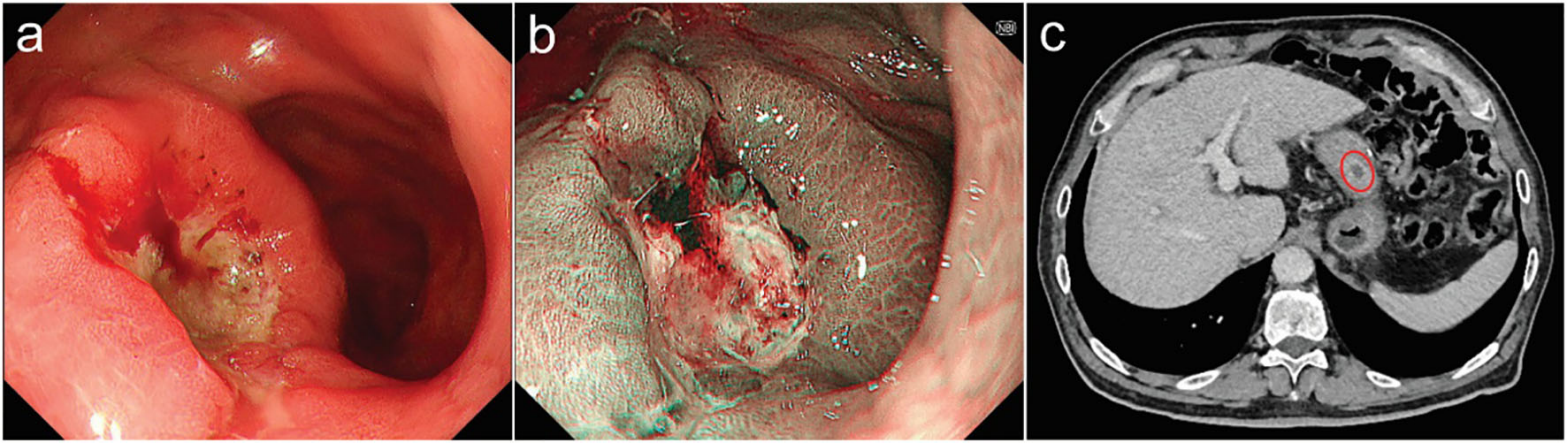
Endoscopic images one year after SASI surgery (July 2025). An ulcerative lesion is visible on the posterior wall of the gastric body. **(a)** Gastroscopic view in standard white-light mode; **(b)** Gastroscopic view in narrow-band imaging (NBI) mode; **(c)** CT images one year after SASI surgery showing localized enhancing wall thickening around the ulcer site (indicated by red circle).

In July 2025, one year after bariatric surgery, the patient underwent routine gastroscopy (**Figures 1a, b**). A deep, depressed ulcerative lesion measuring approximately 1.2 cm × 1.0 cm was identified on the posterior wall of the gastric body. The base was covered with a white coating, and the surrounding mucosa exhibited hyperemia and swelling, indicating an active ulcer. Biopsy results indicated gastric adenocarcinoma with negative Helicobacter pylori status. CT imaging (**Figure 1c**) revealed localized thickening and contrast enhancement of the residual gastric wall. At this time, the patient’s BMI was 29.4 kg/m2, with no anemia or nutrient deficiencies. Tumor markers CEA and CA19–9 remained within normal ranges.

Based on the biopsy-proven gastric adenocarcinoma and imaging findings suggesting localized disease, the patient underwent indocyanine green fluorescence-guided laparoscopic radical gastrectomy. Postoperative pathological examination confirmed moderately to poorly differentiated EBV-associated gastric carcinoma, with tumor invasion extending to the subserosal layer, corresponding to pathological stage IIA (9). Postoperative molecular and MRD assessments were subsequently performed to further characterize the tumor and provide a baseline for longitudinal monitoring. Considering the patient’s compliance and safety, postoperative oral S-1 (Tegafur, Gimeracil and Oteracil Potassium Capsules) chemotherapy was administered (10, 11). The specific regimen consisted of 60 mg daily for 2 consecutive weeks, followed by a 1-week rest period, with each 3-week interval defined as one treatment cycle; the planned treatment duration was 8 cycles.

**Figure 2:**
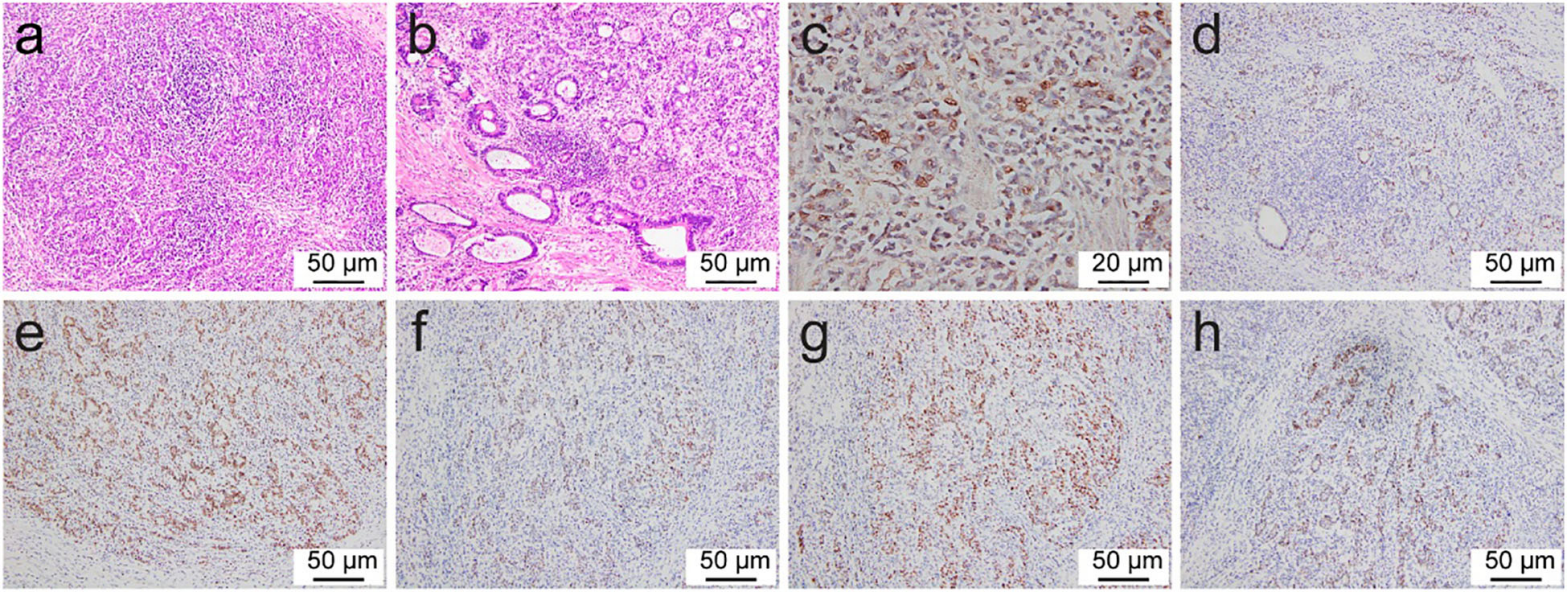
Pathological images of the gastric carcinoma postoperatively. **(a, b)** HE staining; **(c)** EBER *in situ* hybridization: diffuse strong nuclear positivity was observed in tumor cells; **(d)** Immunohistochemistry for p53. **(e)** MLH1(+), **(f)** MSH2(+), **(g)** MSH6(+), **(h)** PMS2(+).

The postoperative pathological examination results of gastric cancer are shown in **Figure 2**. Hematoxylin and eosin (HE) staining revealed infiltrative growth of moderately to poorly differentiated adenocarcinoma. Significant inflammatory cell infiltration was observed in the stroma, along with lymphoid follicle-like structures, suggesting the “lymphoepithelioma-like” background commonly associated with EBV-associated gastric carcinoma. The tumor formed irregular glandular structures accompanied by stromal fibrosis, with dense lymphocyte clusters visible in the surrounding area. EBER *in situ* hybridization confirmed diffuse and strong positivity in the tumor cell nuclei, confirming EBV association. To exclude focal or incidental EBER positivity, EBER-ISH was performed on two formalin-fixed paraffin-embedded tumor blocks representing two separate tumor regions; both areas showed diffuse nuclear positivity in tumor cells, supporting a diffuse EBV-associated pattern. Immunohistochemistry for p53 showed scattered, weak to moderate nuclear staining in tumor cells, consistent with a wild-type expression pattern. Nuclear expression of MLH1, MSH2, MSH6, and PMS2 was retained in tumor cells, with stromal lymphocytes and glandular epithelium serving as well-stained internal controls, suggesting proficient mismatch repair (pMMR)/microsatellite stable (MSS) status. Next-generation sequencing (NGS) of the tumor tissue identified a truncating ARID1A mutation (p.Y215*, variant allele frequency (VAF) 4.56%) and CCND1 amplification (copy number approximately 6). Microsatellite status was MSS. Plasma circulating tumor DNA (ctDNA)/minimal residual disease (MRD) analysis showed no detectable tier I/II tumor-derived variants and was therefore interpreted as MRD-negative at the tested time point. A single ATR p.E275K variant was detected at a low variant allele frequency (VAF 0.10%) and was classified as a variant of uncertain significance (VUS), rather than definitive evidence of molecular residual disease. The ctDNA-equivalent value was 339.4 hGE/mL and was recorded as an exploratory quantitative parameter for longitudinal monitoring, not as a validated cutoff for recurrence risk. A follow-up blood test six months postoperatively showed a comparable ctDNA-equivalent value of 336.4 hGE/mL.

## Patient perspective

The patient was satisfied with the postoperative outcomes after MBS and missed the scheduled 6-month follow-up because he felt well and had no apparent discomfort. After gastric cancer was detected at the 1-year surveillance endoscopy, he actively cooperated with further evaluation, surgery, adjuvant chemotherapy, and follow-up. This experience made the patient recognize that satisfactory postoperative recovery does not eliminate the need for regular follow-up and long-term surveillance.

**Figure 3:**
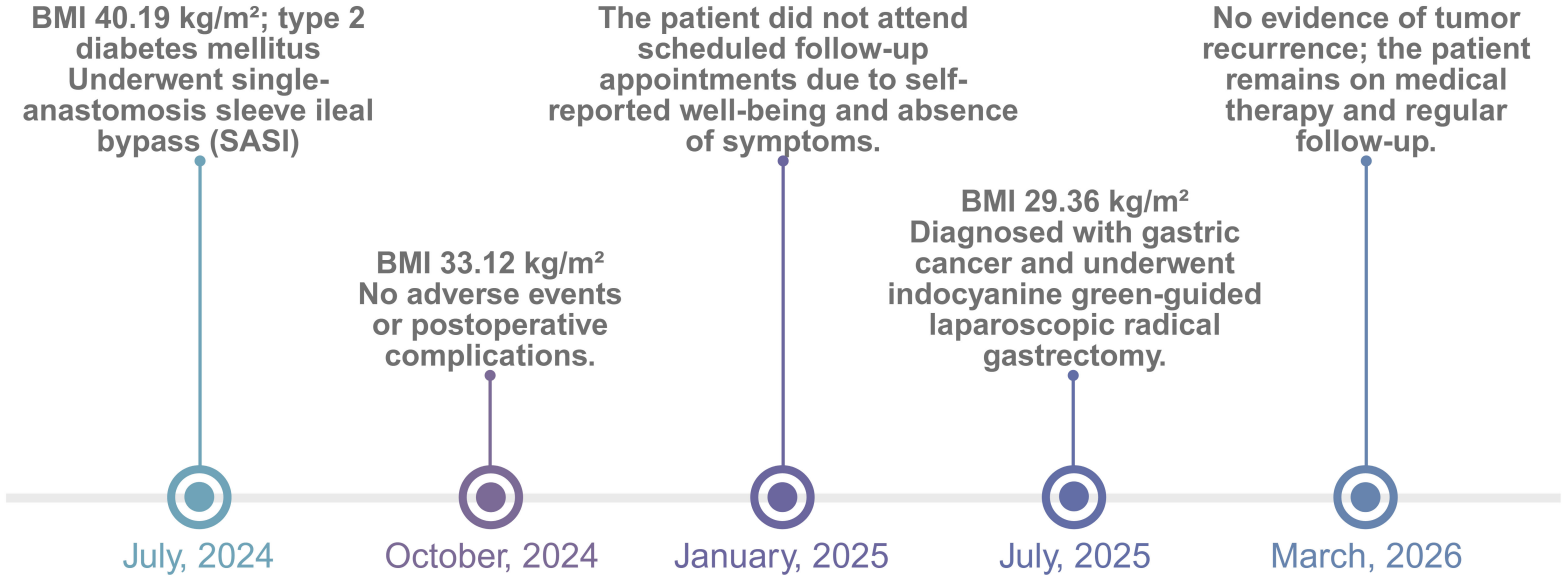
Timeline of the patient’s clinical course, including SASI, postoperative follow-up, missed 6-month visit, 1-year surveillance gastroscopy, biopsy diagnosis, staging CT, radical gastrectomy, pathological and molecular evaluation, adjuvant S-1 chemotherapy, and postoperative ctDNA/MRD monitoring.

Follow-up at eight months postoperatively showed good recovery with no signs of tumor recurrence or metastatic disease. Continued follow-up is ongoing (**Figure 3**).

## Discussion

In this case, serological EBV and *H. pylori* tests were consistently negative at both surgical time points. The discrepancy between serological EBV negativity and histological positivity aligns with the biological characteristics of EBVaGC, which is predominantly characterized by latent infection and low circulating viral load. EBER-ISH remains the gold standard for diagnosis; a prospective study of 2760 patients showed that plasma EBV-DNA is detectable in only half of EBVaGC patients (12). Therefore, negative plasma results do not exclude EBVaGC. While *H. pylori* and EBV co-infection raises cancer risk 2.57-fold (13), the consistent absence of *H. pylori* here suggests an EBV-driven pathway independent of *H. pylori* mediation. EBV is associated with various malignancies and can induce hypermethylation in epithelial cells, particularly during chronic inflammation (14–16).

Gastric cancer after MBS has been reported but remains uncommon. Population-level data from a JAMA Surgery cohort of 908, 849 patients with severe obesity, including 303, 709 who underwent bariatric surgery, did not show an increased risk of esophageal or gastric cancer after bariatric surgery (4). In contrast, case-based evidence remains limited: prior reviews have documented post-bariatric gastric cancers across different procedures, including 22 gastric cancers among 33 esophagogastric neoplasms, 9 gastric cancers among 17 sleeve gastrectomy-associated esophagogastric cancers, 61 published gastric cancer cases in a recent secondary analysis, and 324 post-bariatric esophageal or gastric cancer cases in a scoping review, with adenocarcinoma being the predominant histology (17–20). However, EBV status was generally not reported. The present case is therefore a rare early post-SASI gastric adenocarcinoma with histopathologically confirmed EBV association and H. pylori negativity.

EBV can promote immune evasion by dampening innate antiviral signaling and weakening antigen presentation and effector-cell clearance (21). Viral products, including BPLF1, may interfere with cGAS–STING and RIG-I–MAVS signaling, whereas EBV-positive gastric cancers often show immune-checkpoint-related alterations, such as activation of the PD-L1/PD-L2 axis (22, 23).

Following sleeve gastrectomy, reflux and alterations in the gastric microbiota may cause persistent mucosal irritation. This fosters a microenvironment conducive to EBV-CIMP-like epigenetic remodeling and immune evasion (24). Additionally, obesity-induced chronic inflammation and impaired immune surveillance (25, 26), characterized by persistent macrophage infiltration (27), may further weaken the host’s anti-tumor response. While direct evidence linking MBS to EBVaGC is limited, EBVaGC prevalence in gastric remnant carcinoma is four times higher than in primary non-remnant carcinoma (28). Post-MBS anatomical and physiological alterations—such as reduced gastric acid, altered bile acid dynamics (modulating FXR/TGR5 receptors), and shifts in intestinal permeability—remodel the mucosal barrier and immunity (6–8). This immunometabolic reorganization may provide a “boost” for the transformation of pre-existing latently EBV-infected epithelial clones.

Postoperative ctDNA detection has shown prognostic value in gastric cancer, with ctDNA positivity after curative-intent treatment associated with a higher risk of recurrence and shorter survival (29, 30). However, the clinical interpretation of ctDNA in gastric cancer remains assay-dependent, and standardized thresholds for ctDNA-equivalent values such as hGE/mL have not yet been validated for gastric cancer or EBVaGC (31). Therefore, in the present case, the hGE/mL value was used only as an exploratory baseline for longitudinal monitoring. The absence of detectable tier I/II tumor-derived variants supports an MRD-negative result at the tested time point, whereas the isolated low-level ATR VUS was not considered sufficient to define molecular residual disease. Further prospective studies are needed to establish tumor-specific MRD thresholds, optimal sampling intervals, and clinically actionable ctDNA cutoffs in gastric cancer, particularly in EBVaGC.

Molecularly, an ARID1A p.Y215* nonsense mutation was detected. ARID1A is a frequently inactivated member of the SWI/SNF complex in EBVaGC, occurring against a CIMP-type hypermethylation background (32, 33). CCND1 amplification was also detected. In gastric cancer, CCND1 encodes cyclin D1 and represents a cell-cycle-related alteration. However, TCGA data indicate that EBV-positive tumors are more typically characterized by PIK3CA mutations, extreme DNA hypermethylation, and JAK2/CD274/PDCD1LG2 amplification, whereas CCND1 amplification is more often described within the chromosomal instability subtype (23, 34, 35). Thus, the biological significance of CCND1 amplification in this EBVaGC case remains uncertain. This loss may be mediated by EBV-miR-BARTs, which suppress various host tumor suppressors (36–38). Furthermore, postoperative micronutrient deficiencies (e.g., vitamins A/D, B12, iron) can impair immune function, creating a window for the expansion of focal EBV-latent clones (39). Morphologically, the tumor showed dense lymphocytic infiltration and wild-type p53 expression, consistent with the low TP53 mutation rate in EBVaGC (40). The pMMR/MSS status aligns with the molecular stratification where EBVaGC and MSI-H are typically mutually exclusive. However, the immune-rich microenvironment of EBVaGC often correlates with PD-L1 upregulation, suggesting potential immunotherapy sensitivity (41).

Taken together, this case suggests that EBVaGC presenting as “histologically positive but serologically negative” is biologically plausible within the context of post-MBS immunometabolic remodeling. Latent EBV infection, obesity-related chronic inflammation, and post-MBS anatomical and physiological changes may converge to create a permissive mucosal microenvironment that promotes EBV-CIMP-like epigenetic remodeling, ARID1A inactivation, and immune evasion, thereby facilitating the malignant transformation of EBV-latently infected gastric epithelial clones without significant viremia. Consequently, follow-up strategies are crucial. For post-MBS patients with persistent reflux, nausea, anemia, or unexplained hypoalbuminemia, the threshold for diagnostic endoscopy should be lowered, and routine gastroscopy approximately one year after MBS may be considered to assess the gastric remnant and mucosal health.

## Conclusion

This case involves an EBV-associated gastric adenocarcinoma that occurred 12 months after SASI, characterized by “histological EBER-ISH positivity with negative serum/plasma EBV, and negative *H. pylori* status at two time points. This case supports the biological model that postoperative anatomical and immune-metabolic remodeling can promote the tumorigenesis of focal latent EBV epithelial clones without requiring systemic viremia. It emphasizes the importance of adhering to histological EBV testing and performing molecular stratification when upper gastrointestinal alarm symptoms appear after MBS, alongside incorporating nutritional management and accessible endoscopic follow-up to promptly correct nutritional deficiencies. This case provides empirical evidence for the identification and management of EBVaGC in the MBS population and suggests the necessity of prospective studies to optimize postoperative screening and follow-up strategies.

## Data Availability

The original contributions presented in the study are included in the article/supplementary material. Further inquiries can be directed to the corresponding author.
